# Dietary Calcium Intake and Osteoporosis Risk in Arab Adults

**DOI:** 10.3390/nu15132829

**Published:** 2023-06-21

**Authors:** Nasser M. Al-Daghri, Syed Danish Hussain, Abdullah M. Alnaami, Naji Aljohani, Shaun Sabico

**Affiliations:** 1 Chair for Biomarkers of Chronic Diseases, Biochemistry Department, College of Science, King Saud University, Riyadh 11451, Saudi Arabia; 2 Obesity, Endocrine and Metabolism Center, King Fahad Medical City, Riyadh 12231, Saudi Arabia; 3 College of Medicine, Alfaisal University, Riyadh 11533, Saudi Arabia

**Keywords:** osteoporosis, dietary intake, bone mineral density

## Abstract

Osteoporosis is a major public health concern in Saudi Arabia’s aging population. There is particularly limited information on how diet affects bone loss in this ethnic group. The purpose of this study was to examine the association between dietary calcium (Ca) intake and osteoporosis risk in Saudi adults. A total of 1950 patients (416 males and 1534 females) with known risk factors for osteoporosis participated in this cross-sectional study. A short questionnaire (CaQ) was used to assess dietary Ca intakes in patients attending tertiary hospitals in Riyadh City. The prevalence of osteoporosis was 21.3% and was more common in females (93.5%). Patients with osteoporosis were older (*p* < 0.001) and had lower BMI (*p* < 0.001). Results showed that the overall mean Ca intake was only 445.1 mg/day (recommended dietary intake of 1300 mg/day). Tea intake (OR = 0.8 95%CI: 0.7–1.0; *p* = 0.02) and consumption of fish and eggs (OR = 0.9 95%CI: 0.8–1.0; *p* = 0.01) were significantly associated with a lower risk of osteoporosis. Furthermore, consumption of biscuits, cake and bread slices were significantly associated with higher incidence of osteoporosis (OR = 1.3 95%CI: 1.0–1.5; *p* = 0.02). In conclusion, extremely low dietary Ca intake was observed among Saudi adults already at risk of osteoporosis. A balanced diet including high amount of Ca, vitamin D and omega-3 fatty acids accompanied by limiting consumption of foods high in saturated fats and glycemic index may be helpful in reducing osteoporosis risk in the Saudi adult population.

## 1. Introduction

Osteoporosis is a common disease in Saudi Arabia (SA), resulting from decreased bone mass, strength and microarchitecture, which ultimately elevates the risk of fractures [1]. The World Health Organization (WHO) defined osteoporosis as a decrease in bone mineral density (BMD), measured by dual-energy X-ray absorptiometry (DXA), to at least 2.5 standard deviations below the mean peak BMD of a young adult woman [2]. In SA, the most recent hospital-based study showed that the prevalence of osteoporosis was as high as 64% in men and 58% in post-menopausal women above 50 years old [3]. Factors contributing to the high prevalence of osteoporosis in SA include advancing age, history of previous fracture, family history of low bone mass, menopause and use of corticosteroids [4].

Individuals diagnosed with osteoporosis have a 40% risk of fractures, which can result in mobility impairment and significantly reduce quality of life [1]. Available evidence suggests that bone loss related to age and/or post-menopause is influenced by the level of peak bone mass attained during childhood and adolescence, as well as the rate at which bone is lost [5]. Genetic factors can also explain between 60% and 80% of the variations in peak bone mass, with the remainder modulated by external factors such as nutrition [5,6,7]. In fact, consuming sufficient dietary calcium (Ca) and protein are necessary to attain ideal peak bone mass and prevent bone loss in adults [8].

Diet plays a significant role in the prevention and management of osteoporosis [8]. Consuming a diet that is rich in Ca, vitamin D, vitamin K and magnesium, and a balanced intake of protein, fruits, milk, yogurt, cheese, dark leafy green vegetables, such as spinach and kale, fish such as sardines, eggs and fortified foods such as breakfast cereals can contribute to good bone health by promoting bone formation and reducing bone loss [9,10,11,12,13]. However, cakes, biscuits and other foods that are high in added sugars and refined grains do not provide the same benefits for bone health and may be associated with an increased risk of inflammation and bone loss over time [7,8,9]. 

Several studies have investigated the association of dietary patterns with osteoporosis and BMD in different ethnic groups and have reported mixed results [14,15,16]. In contrast, there are limited studies conducted in Arabian populations, particularly in SA, as the available literature has focused mainly on osteopenia in post-menopausal women, extra-skeletal effects of vitamin D and more recently, biochemical osteomalacia in Arab adolescents [17,18,19]. In order to fill this gap, this study examined whether dietary patterns, and Ca intake in particular, are associated with osteoporosis risk in Saudi Arabian adults.

## 2. Materials and Methods

This multi-center cross-sectional study was taken from the Osteoporosis Registry database of the Chair for Biomarkers of Chronic Diseases (CBCD) in King Saud University (KSU), SA, in collaboration with the Ministry of Health. The project received approval from the Ethics Committee at the College of Medicine, KSU, Riyadh, SA (number H-01-R-012). In brief, 2185 records of all consenting Saudi adults visiting the major tertiary hospitals in Riyadh (King Fahad Medical City (KFMC), King Khalid University Hospital (KKUH) and King Salman Hospital (KSH)) who were advised to have their BMD assessed from 2013–2016 were included [20]. 

### 2.1. Study Design and Participants

**Figure 1:**
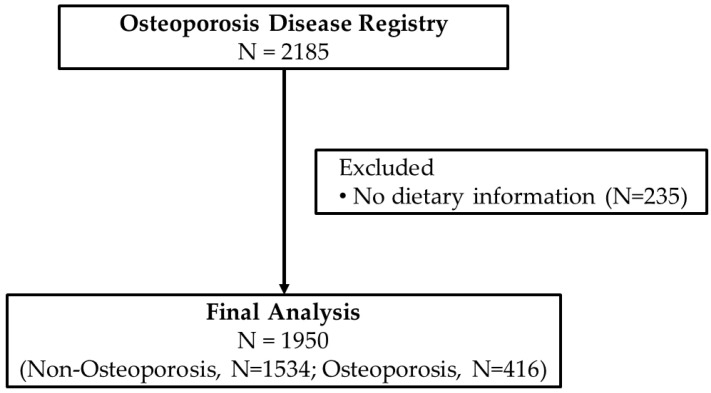
Flowchart of participants.

Data collection for the Osteoporosis Registry started in 2013 and ended in August 2016. Screening of participants for inclusion in the registry were overseen by trained physicians in their respective radiology departments in participating hospitals where a BMD (DXA) scan is available. The inclusion criteria were those preferably, but not limited to, post-menopausal women, those with history of fracture in the last 5 years, diminished height in the last 2 years, men and women with history of malabsorption and nutritional deficiencies (vitamin D and Ca), who had endocrine disorders affecting skeletal mass (e.g., hyperparathyroidism), were at risk for bone density loss (family history of osteoporosis) and had an available BMD scan. All participants were asked for their consent before inclusion in the registry. Participants were excluded if they were <35 years of age or if they have any malignancy, cardiac disorders, lung disease or any disease that required immediate medical attention. For this particular study, participants were excluded if there were no dietary data available. Figure 1 shows the flowchart of participants. 

### 2.2. Diagnosis of Osteoporosis

Diagnosis of osteoporosis was undertaken by measuring BMD through a DXA scan of the femoral neck or spine and calculating the T-score. A patient with a T-score of ≤−2.5 (standard deviations, SD, from the mean value of young normal Caucasian women) is classified as having osteoporosis [21,22]. DXA scans were overseen by a qualified technician certified in densitometry, while the machine itself was calibrated using a standard phantom provided by the manufacturer. Upon completion of the test, the BMD findings and corresponding T-scores were forwarded to the Chair for Biomarkers in Chronic Diseases (CBCD) at King Saud University, SA, for classification according to osteoporosis status and data entry.

### 2.3. Anthropometry, Blood Collection and Sample Analysis

Clinical data were obtained by trained nurses using standard techniques to measure anthropometrics which included height (cm), waist/hip measurements (cm) and weight (kg). Body mass index (BMI) was computed as weight (in kilograms) divided by height (in squared meters). Blood pressure (mmHg) was recorded twice, with a 10-min interval between readings, using a mercurial sphygmomanometer, and the average reading was noted. Fasting blood samples were extracted and subsequently sent to the CBCD laboratory for biochemical analysis using an automated biochemical analyzer (Konelab 20, Thermo-Fischer Scientific, Espoo, Finland). This analysis included serum lipid profiling as well as quantification of fasting glucose, Ca and albumin levels.

### 2.4. Dietary Intake 

Participants were invited for the administration of a short questionnaire (CaQ) adopted from Macdonald et al. [23]. The CaQ is a validated instrument to assess daily dietary Ca intakes [23]. The information collected was on weekly consumption of food and beverages that are common sources of Ca in SA which included milk, tea with milk, coffee with milk, bread, biscuits, cakes, eggs, fish, cheese, yogurt, green vegetables, porridge, pancakes and lasagna. The participants were asked to provide weekly standard servings of the mentioned foods and beverages. Then, weekly Ca intake was calculated by multiplying the amount of Ca present in the standard serving of the food/beverage. 

### 2.5. Data Analysis

Data were analyzed using SPSS, version 21.0. The continuous variables were expressed as the mean with the standard deviation or as the median with the quartile range. The categorical variables were presented as frequencies and percentages. To determine significance, the independent sample *t*-test and Mann–Whitney *U* test were used for normal and non-normal variables, respectively. Age and BMI were adjusted for using univariate analysis of variance. Prior to conducting parametric testing, non-normal variables were log-transformed. Multivariate logistic regression analysis was used to obtain both unadjusted and adjusted odds ratios (OR). A correlation matrix was performed among different sources of dietary Ca prior to univariate modelling to confirm absence of multicollinearity. Significance was set at *p* < 0.05.

## 3. Results

A total of 1950 patients (N = 1534 (389 males and 1145 females) without osteoporosis and N = 416 (27 males and 389 females with osteoporosis)) were included in this observational study. The prevalence of osteoporosis was 21.3%, of which 93.5% were females. Patients with osteoporosis were significantly older (*p* < 0.001) and had lower BMI (*p* < 0.001). Table 1 shows the anthropometrics, clinical characteristics and biochemical parameters according to osteoporosis status.

After adjusting for age and BMI, the osteoporosis group appeared to have significantly lower systolic blood pressure (*p* = 0.05), diastolic blood pressure (*p* = 0.005), glucose level (*p* = 0.01) and triglycerides (*p* = 0.001) than the non-osteoporosis group. However, the osteoporosis group have significantly higher 25(OH)D concentrations than the non-osteoporosis group (*p* < 0.001).

**Figure 2:**
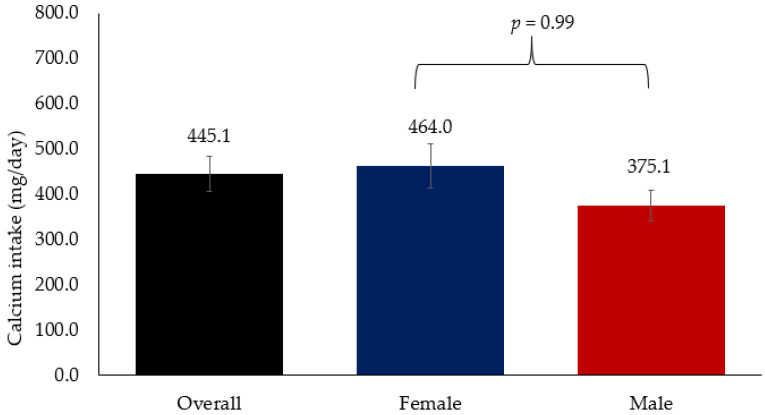
Mean Ca intake among Arab adults.

Figure 2 shows the overall mean Ca intake which was 445.1 mg/day, which is far below the recommended dietary intake of 1300 mg/day. Mean Ca intake among women was 464.0 mg/day while in men it was 375.1 mg/day. No significant difference in Ca intake was observed between men and women. 

Table 2 shows the Ca intake from food groups according to osteoporosis status. The Ca intake from tea with milk was significantly higher in the non-osteoporosis group than the osteoporosis groups with odds ratio (OR) of 0.7 (0.6–0.8) (*p* < 0.001). Similarly, the Ca intake of coffee with milk was significantly higher in the non-osteoporosis group than the osteoporosis group with an OR 0.6 (0.5–0.8) (*p* < 0.001). Furthermore, Ca intake from fish and eggs was also significantly higher in the non-osteoporosis group than the osteoporosis group with an OR 0.8 (0.7–0.9) (*p* < 0.001). Ca intake from porridge and oats was also significantly higher in the non-osteoporosis group than osteoporosis group with OR 0.8 (0.8–0.9) (*p* = 0.005). On the other hand, Ca intake from biscuits, cake and bread slices were significantly higher in the osteoporosis group than the non-osteoporosis group (*p* < 0.001). After adjusting for age and BMI, only consumption of tea with milk, biscuits, cake and bread slices and fish and eggs remained significantly different between groups, while consumption of coffee with milk showed borderline significance (*p* = 0.05).

Table 3 shows the odds of osteoporosis according to Ca intake from different food groups obtained from multivariate logistic regression analysis. All these variables were log transformed and added simultaneously to the logistic model adjusting for one another. Results showed that consuming tea (*p* = 0.005), coffee with milk (*p* = 0.007) and fish and eggs (*p* = 0.002) were significantly associated with lower risk of osteoporosis. After adjustment with age and BMI, consumption of biscuits, cake and bread slices remained significantly associated with higher risk of osteoporosis (*p* = 0.02). 

## 4. Discussion

The present study aimed to determine the association between dietary Ca intake and osteoporosis risk in Saudi adults with known risk factors for osteoporosis. Results showed that the overall mean Ca intake, which was 445.1 mg/day, was substantially below the recommended dietary intake of 1300 mg/day. Mean Ca intake of women was 464.0 mg/day, while in men it was 375.1 mg/day. Dietary Ca intake from tea with milk, fish and eggs were significantly associated with lower risk of osteoporosis while biscuits, cake and bread slices showed a significant association with higher risk of osteoporosis independent of age and BMI.

Ca is an essential divalent cation that is mainly present outside cells and plays a major role in a multitude of vital functions such as muscle contraction, neuronal excitability, neurotransmitter release and blood coagulation. Additionally, the Ca reservoir significantly contributes to the mineral matrix of bone, an indication that the reservoir can influence bone strength [24] and serve as a buffer when required [25]. It is well-established that consuming enough Ca during the period of growth is the most significant nutritional factor for attaining optimal peak bone mass [25,26,27]. A 2017 systematic review found that the average Ca intake in national diets ranged from 175 to 1233 mg/day, as reported in 78 studies from 74 countries [28]. Similar to the present study, the average dietary Ca intake in many Asian countries is less than 500 mg/day, while countries in Africa and South America generally ranged from 400 to 700 mg/day [28]. 

Additionally, a more recent systematic review was conducted on the diets of individuals aged 25 and older in 195 countries, which used national or sub-national representative nutrition surveys and multiple dietary data sources from the Global Health Data Exchange [29]. According to this review, the global average daily Ca intake is approximately 400 mg/day, with lower intakes observed in Sub-Saharan African countries and Southeast Asia (around 200 mg/day) as compared to high-income countries (around 600–800 mg/day) [29]. In this study, the average Ca intake in adult males was 375.1 mg/d, while for females it was 464.0 mg/d. Unfortunately, inadequate Ca intake and suboptimal levels of vitamin D are prevalent among the elderly population of SA, with the latter improving over time across all demographic groups due to successful public health campaigns [30]. It is expected that this improvement in vitamin D levels may also bring a general improvement in Ca intake through continuous public health campaigns. 

This study suggests that Ca intake from tea with milk is significantly associated with decreased risk of osteoporosis. Some studies reported that frequent tea and coffee intake had little influence on BMD and were not associated with fracture risk among postmenopausal women with osteoporosis [31,32]. In a meta-analysis which included 17 studies [14], the odds of osteoporosis for the highest versus the lowest categories of tea consumption was 0.62 (95%CI, 0.46–0.83). Furthermore, the risk-lowering effect of tea consumption on osteoporosis was consistent in males and females, different study periods, study designs and multiple geographic locations [14]. This beneficial effect of tea consumption with milk may be due to the presence of polyphenols that are responsible for improving BMD by reducing oxidative stress and inflammation [33]. Additionally, polyphenols are known to be sequestered into physiological bone, which may help protect it from degradation [14,33]. While more research is needed to understand the exact mechanisms by which polyphenols may help protect bone health, it is suggested that consuming polyphenol-rich foods, such as tea, may help protect against bone loss.

The present study also found the protective effects of fish and eggs consumption on osteoporosis risk. Both fish and eggs are known to be great sources of protein which help build and maintain strong bones and muscles. Pujia et al. [17] also reported a positive association between whole egg consumption and bone health. Furthermore, numerous studies have shown that fish consumption is linked to decreased osteoporosis risk, increased BMD and decreased fracture risk for both men and women—more so in Asian populations [34,35,36,37] than Caucasians, as observed in the Cardiovascular Health Study [38]. 

In the present study, Ca intake from cake, biscuits and bread contributed to increased osteoporosis risk. These results are consistent with other observations which found that high glycemic index (GI) foods had negative consequences for bone health, including decreased osteoblast activity, increased bone resorption, oxidative stress and inflammation, as well as the induction of acidosis [39,40]. In contrast, complex carbohydrates such as those found in vegetables, legumes and whole grains can increase Ca absorption and neutralize metabolic acid loads in the body [9]. Inulin-type fructans are a form of non-digestible carbohydrate found in various vegetables and fruits, which cannot be broken down by mammalian enzymes [9]. Inulin-type fructans alter intestinal Ca absorption, enhance Ca solubility in the lumen and amplify bioavailability, eventually leading to improved passive transport of into the body [9,41]. While the present study did not observe an association between vegetable and fruit consumption on osteoporosis risk, it should not supersede previous findings on the beneficial effects of vegetables and fruits consumption on bone health [41,42,43]. 

Large populations across the globe consider milk and dairy products, including cheese, butter, cream and yogurt, to be major sources of Ca, which can increase BMD and potentially reduce the risk of fractures, particularly in postmenopausal women [15,40,44]. A recent 2021 systematic review of 8 case-control studies, 10 cross-sectional studies, 17 prospective cohort studies and 14 randomized clinical trials (RCT) reported that dairy consumption had a moderate effect on bone health in older adults aged 50 years or more [44]. All case-control studies showed reduced hip fracture risk with the exception of one study; the included cross-sectional studies showed favorable impact of milk intake on BMD; and RCTs showed a beneficial impact of fortified dairy products [44]. In the same review, however, there is ambiguity as to the life period in which milk intake is most advantageous [44].

It is important to note that the relationship between dairy intake and bone health is also influenced by other factors such as exercise, genetics and overall nutrition [45,46]. For instance, hip fracture risk in the elderly was found to be higher in those who self-reported consuming milk and dairy products, specifically at the age of 20 years, suggesting a potential negative impact of high dairy intake [46]. In Feskanich et al.’s investigation, Ca supplements may help prevent osteoporotic fractures in certain bone areas, but regularly consuming high amounts of Ca-rich foods during middle age is unlikely to confer any protective benefits against hip or forearm fractures, at least for white middle-aged women in the US [45]. The mentioned study was followed-up after 6 years and involved analysis of 72,337 postmenopausal women where a total of 603 hip fractures occurred due to low or moderate trauma during the period from 1980 to 1998 [47]. The risk of hip fracture was not linked to either milk intake or total Ca consumed, and instead there was a lower risk of osteoporotic hip fractures in postmenopausal women with sufficient intake of vitamin D [47]. The results of our study also showed no association between Ca intake from dairy products and risk of osteoporosis. These inconsistencies in the research regarding a potential link between milk consumption and bone health can be attributed, in part, to the biases and inaccuracies inherent in dietary data collection [48]. 

The present study has certain limitations which should be taken into consideration when interpreting findings. First, the results are based on observational study; thus, causality cannot be inferred. Second, Ca intake from supplements and medications in general were not recorded and this could have explained why the osteoporosis group appeared to have a better metabolic profile (e.g., lower BP and glucose, higher 25(OH)D) than the non-osteoporosis group. Finally, the findings of the present study are most relevant to Arab adults already at risk of osteoporosis. Similar studies using other populations (e.g., pregnant, pre-menopausal) in the same ethnic group may be able to provide additional information on the over-all dietary deficiencies present in the modern-day Saudi diet.

## 5. Conclusions

In conclusion, extremely low dietary Ca intake was observed among Saudi adults at risk of osteoporosis. Ca intake from tea with milk, fish and eggs decrease, while Ca intake from cakes, biscuits and bread increase, the risk of osteoporosis. These findings suggest an urgent need to promote Ca supplementation in this high-risk population since diet alone is clearly not enough to meet daily Ca needs. A balanced diet that meets the recommended intake of Ca, vitamin D and omega-3 fatty acids, as well as limiting foods high in saturated fats and/or glycemic index, may be effective in reducing osteoporosis risk in the Saudi adult population.

## Figures and Tables

**Table 1:** Anthropometrics and biochemical parameters according to osteoporosis status.

Parameters	Non-Osteoporosis	Osteoporosis	*p*-Value	*p*-Value *
N	1534	416		
Age (years)	53.3 ± 11.5	59.3 ± 8.7	<0.001	--
BMI (kg/m^2^)	32.3 ± 6.2	30.3 ± 6.1	<0.001	--
M/F	389/1145	27/389	<0.001	--
T2DM	725 (47.3)	193 (46.4)	0.30	0.06
Hypertension	547 (35.7)	128 (30.8)	0.06	0.01
WHR	0.9 ± 0.4	0.9 ± 0.2	0.40	0.26
Systolic BP (mmHg)	125.3 ± 16.4	124.4 ± 17.8	0.36	0.05
Diastolic BP (mmHg)	77.5 ± 10.0	76.2 ± 10.3	0.02	0.005
Total Cholesterol (mmol/L)	5.1 ± 1.2	5.1 ± 1.1	0.64	0.57
Glucose (mmol/L)	6.4 (5.3–9.5)	6.2 (5.3–8.8)	0.76	0.01
HDL-C (mmol/L)	1.1 ± 0.4	1.2 ± 0.4	0.18	0.12
Triglycerides (mmol/L)	1.6 (1.2–2.2)	1.5 (1.1–2.0)	0.006	0.001
25(OH) D (nmol/L)	59.4 (36.9–89.1)	72.2 (44.8–100.1)	<0.001	0.09
Ca (mmol/L)	2.3 ± 0.3	2.3 ± 0.3	0.16	0.49
Phosphorous (mmol/L)	1.3 (1.0–1.7)	1.2 (1.0–1.7)	0.61	0.50
BMD Spine	1.0 ± 0.3	0.8 ± 0.2	<0.001	<0.001
BMD Femur	1.0 ± 0.1	0.9 ± 0.2	<0.001	<0.001
T-score Spine	−0.8 ± 1.2	−3.1 ± 0.6	<0.001	<0.001
T-score Femur	−0.1 ± 2.9	−1.3 ± 0.9	<0.001	<0.001
Tea with Milk (cups/wk)	0.8 ± 2.9	0.7 ± 3.3	<0.001	0.53
Coffee with milk (cups/wk)	0.5 ± 2.9	0.2 ± 1.0	<0.001	0.12
Slices (Count/wk)	5.6 ± 7.5	6.5 ± 6.9	<0.001	0.42
Biscuits (Count/wk)	0.9 ± 2.9	0.7 ± 2.2	0.06	0.92
Cake (Portion/wk)	0.8 ± 4.7	0.6 ± 3.0	0.001	0.33
Pancakes (Count/wk)	0.3 ± 3.3	0.2 ± 0.7	0.40	0.99
Lasagna (Portion/wk)	1.0 ± 11.2	0.8 ± 10.4	0.45	0.76
Whole meal Slices (Count/wk)	0.6 ± 2.2	0.5 ± 1.7	0.19	0.38
Milky drinks (Count/wk)	2.7 ± 5.7	2.9 ± 6.4	0.44	0.67
Milk with cereal (Portion/wk)	0.2 ± 1.6	0.1 ± 0.6	0.15	0.95
Cheese (Triangle/wk)	2.5 ± 11.3	2.5 ± 8.8	0.20	0.48
Milk pudding (Portion/wk)	0.4 ± 3.7	0.2 ± 1.5	0.001	0.16
Cottage Cheese (Portion/wk)	1.5 ± 6.0	1.4 ± 6.5	0.45	0.16
Sardines pilchards (Portion/wk)	0.9 ± 7.9	0.5 ± 6.0	0.002	0.05
Fish (Portion/wk)	1.1 ± 7.9	0.7 ± 4.7	0.04	0.87
Eggs (Eggs/wk)	1.2 ± 3.7	0.8 ± 2.4	<0.001	0.60
Cheese omelet (Count/wk)	0.4 ± 6.0	0.5 ± 6.2	0.16	0.83
Porridge (Portion/wk)	0.2 ± 2.1	0.2 ± 0.6	0.87	0.98
Muesli (Portion/wk)	0.9 ± 5.3	0.8 ± 5.6	0.001	0.97
Green vegetable (Portion/wk)	3.5 ± 5.9	3.9 ± 6.8	0.08	0.20

Note: Data presented as N (%) for frequencies; mean ± SD for normal variables and median (quartile 1–quartile 3) for non-normal variables; *p* < 0.05 considered significant. * indicates *p*-values adjusted for age, BMI and gender.

**Table 2:** Ca intake from different food groups according to Osteoporosis status.

Parameters	Non-Osteoporosis		Osteoporosis		Univariate	Adjusted *	*p*-Value	*p*-Value *
	Mean ± SD	Median (Q1–Q3)	Mean ± SD	Median (Q1–Q3)	OR (95%CI)	OR (95%CI)		
Tea with Milk	33.6 ± 117.2	0.0	26.4 ± 132.7	0.0	0.7 (0.6–0.8)	0.8 (0.6–0.9)	<0.001	<0.001
Coffee with Milk	23.9 ± 144.1	0.0	8.5 ± 50.7	0.0	0.6 (0.5–0.8)	0.8 (0.6–1.0)	<0.001	0.05
Biscuits, cake, bread slices	479.5 ± 2920.0	240.0 (110.0–350.0)	432.3 ± 2618.1	260.0 (150.0–360.0)	1.1 (0.9–1.3)	1.2 (1.0–1.5)	0.30	0.02
Milk and milk products	1627.9 ± 4794.6	1000.0 (335.0–2000.0)	1641.3 ± 4389.7	975.0 (320.0–1850.0)	1.0 (0.9–1.1)	1.0 (0.9–1.1)	0.78	0.61
Green vegetables	140.8 ± 235.5	120.0 (40.0–200.0)	154.5 ± 272.4	120.0 (40.0–200.0)	1.1 (1.0–1.3)	1.1 (1.0–1.3)	0.13	0.15
Fish and eggs	601.4 ± 4720.7	50.0 (0.0–222.0)	457.5 ± 5244.5	37.0 (0.0–100.0)	0.8 (0.7–0.9)	0.8 (0.8–0.9)	<0.001	0.001
Porridge and oats	96.4 ± 553.1	0.0 (0.0–100.0)	80.8 ± 561.9	0.0 (0.0–37.0)	0.8 (0.8–0.9)	1.0 (0.8–1.1)	0.005	0.37

Note: Data presented as ca intake from different food groups (mg/wk); OR obtained from logistic linear regression; * indicates *p*-value adjusted for age and BMI.

**Table 3:** Odds of Osteoporosis according to Ca intake from food groups.

	Model 1		Model 2	
	OR (95%CI)	*p*-Value	OR (95%CI)	*p*-Value
Tea with Milk	0.8 (0.7–0.9)	0.005	0.8 (0.7–1.0)	0.02
Coffee with Milk	0.7 (0.6–0.9)	0.007	0.8 (0.6–1.1)	0.18
Biscuits, Cakes, Pancakes, Bread	1.1 (0.9–1.4)	0.22	1.3 (1.0–1.5)	0.02
Milk and Milk products	1.0 (0.9–1.1)	0.83	0.9 (0.8–1.1)	0.30
Green vegetables	1.2 (1.0–1.4)	0.08	1.1 (0.9–1.3)	0.32
Fish and Eggs	0.8 (0.8–0.9)	0.002	0.9 (0.8–1.0)	0.01
Porridge, Muesli and Oats	0.9 (0.8–1.0)	0.18	1.0 (0.9–1.1)	0.94

Note: Data presented as OR (95%CI); Model 1 include results of multivariate logistic regression; Model 2 include results adjusted for age and BMI; *p* < 0.05 considered significant.

## Data Availability

The data presented in this study are available on reasonable request from the corresponding author.
